# Modern dust aerosol availability in northwestern China

**DOI:** 10.1038/s41598-017-09458-w

**Published:** 2017-08-18

**Authors:** Xunming Wang, Hong Cheng, Huizheng Che, Jimin Sun, Huayu Lu, Mingrui Qiang, Ting Hua, Bingqi Zhu, Hui Li, Wenyong Ma, Lili Lang, Linlin Jiao, Danfeng Li

**Affiliations:** 1 Key Laboratory of Water Cycle and Related Land Surface Processes, Institute of Geographic Sciences and Natural Resources Research, Chinese Academy of Sciences, Beijing, 100101 China; 2 University of Chinese Academy of Sciences, Beijing, 100049 China; 3 State Key Laboratory of Earth Surface Processes and Resource Ecology, Beijing Normal University, Beijing, 100875 China; 4 State Key Laboratory of Severe Weather (LASW), Institute of Atmospheric Composition, Chinese Academy of Meteorological Sciences, Beijing, 100081 China; 5 Key Laboratory of Cenozoic Geology and Environment, Institute of Geology and Geophysics, Chinese Academy of Sciences, Beijing, 100029 China; 6 School of Oceanographic and Geographic Sciences, Nanjing University, Nanjing, 210023 China; 7 Key Laboratory of Western China’s Environmental Systems (Ministry of Education), College of Earth and Environmental Sciences, Lanzhou University, Lanzhou, 730000 China; 8 Key Laboratory of Desert and Desertification, Cold and Arid Regions Environmental and Engineering Research Institute, Chinese Academy of Sciences, Lanzhou, 730000 China

## Abstract

The sources of modern dust aerosols and their emission magnitudes are fundamental for linking dust with climate and environment. Using field sample data, wind tunnel experiments and statistical analysis, we determined the contributions of wadis, gobi (stony desert), lakebeds, riverbeds, and interdunes to modern dust aerosol availability in the three important potential dust sources including the Tarim Basin, Qaidam Basin, and Ala Shan Plateau of China. The results show that riverbeds are the dominant landscape for modern dust aerosol availabilities in the Qaidam Basin, while wadis, gobi, and interdunes are the main landscapes over the Ala Shan Plateau and Tarim Basin. The Ala Shan Plateau and Tarim Basin are potential dust sources in northwestern China, while the Qaidam Basin is not a major source of the modern dust aerosols nowadays, and it is not acting in a significant way to the Loess Plateau presently. Moreover, most of modern dust aerosol emissions from China originated from aeolian processes with low intensities rather than from major dust events.

## Introduction

Modern dust aerosols generated by aeolian processes^1^ play important roles in climate and weather processes^2, 3^, provide nutrient and essential elements for terrestrial and marine ecosystems^4–8^, become dominant sources of loess^9, 10^ and marine sediments^11–13^, and thereby contribute significantly to global climate, carbon and biogeochemical cycles^14, 15^. On global scale, annual intensities of 0.1–10 µm fraction of dust vary between 981 and 4313 Tg^16^, or approximately 1654 Tg yr^−1^ ^17^ or 1490 Tg yr^−1^ ^18^, and the 0.1–6 µm fractions range from 1604 to 1960 Tg yr^−1^ ^19, 20^. Without considering the contributions of human activities, modern dust aerosols emissions originating from natural sources are 1840 Tg yr^−1^ ^21^. These large differences may result in uncertainties in reconstructing and predicting climate and environmental changes of the past and present, determining current air pollution controls, and combating desertification.

**Figure 1:**
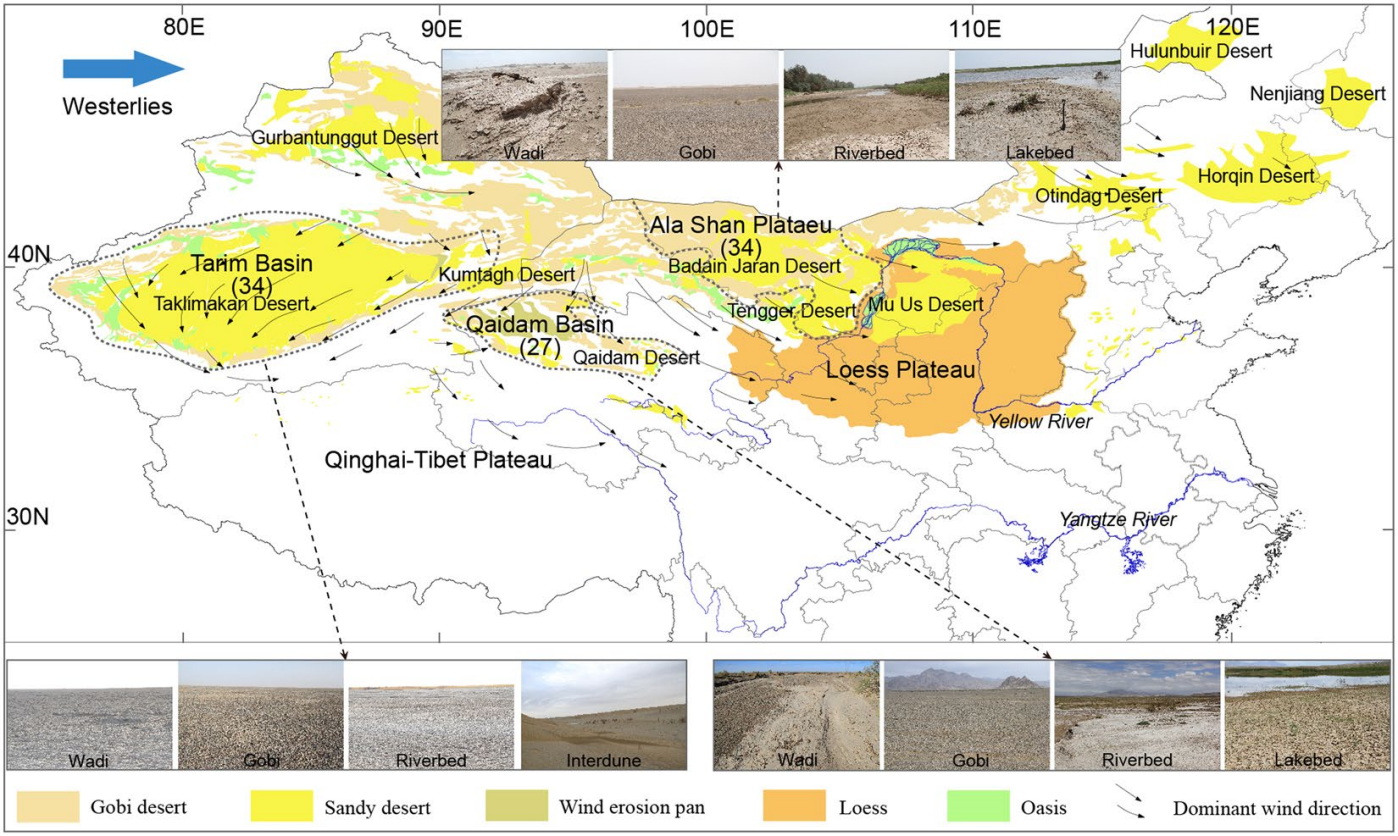
Locations of the Qaidam Basin, Ala Shan Plateau, Tarim Basin, and the landscapes with high modern dust aerosol emissions. Numbers within the brackets are the number of samples. The figure was finished using Arcgis software (version 10.1, ESRI Inc., Redlands, California, USA), which can be downloaded from the internal network of Institute of Geographic Sciences and Natural Resources Research, Chinese Academy of Sciences.

In Asia, approximately 400 to 1100 Tg yr^−1^ of modern dust aerosols, or more than 50% of the global total, originate from China such as from the Qaidam Basin, Tarim Basin and Ala Shan Plateau^22–26^ (Fig. 1). They are transported over large areas of Asia and across the Pacific to North America^27–30^, and the potential landscapes of modern dust aerosol emissions may include gobi^31^, sandy deserts^22, 32–35^ and other landscapes, such as riverbeds, lakebeds, and wadis (dry ancient lakebed /riverbed that contains water only during the periods of heavy rain or simply an intermittent stream)^36, 37^. However, the heterogeneity of underlying surfaces may result in large differences in aeolian process intensities^38, 39^, leading to uncertainties for estimating modern dust aerosol emission quantities and identifying the potential modern dust aerosol source areas in the region.

The amount of fine materials (i.e., <50 µm in diameter) transported by aeolian processes is taken as the dust aerosol availability^40^, which is important for understanding the intensity of dust aerosol loading in the region^41^. Although there have been direct measurements of aerosol loadings in Chinese desert regions^22, 42^, the absence of direct measurements of modern dust aerosol availabilities in different landscapes gives rise to several uncertainties (*S1*), especially when 1) simulating global and regional modern dust aerosol emission intensities; 2) assessing the impacts of modern dust aerosols on global climate, carbon and biogeochemical cycles; 3) identifying the source of aeolian sediments that have been employed as proxies in climate reconstructions in the sediment areas^43, 44^. To address this gap, we use field investigations, sampling, wind tunnel experiments, and particle size measurements as well as the statistical analysis to determine modern dust aerosol availabilities of different landscapes over the Tarim Basin, Qaidam Basin and Ala Shan Plateau. The results improve our understanding of modern dust aerosol availabilities and aeolian sediment sources in China.

## Results and Discussion

**Figure 2:**
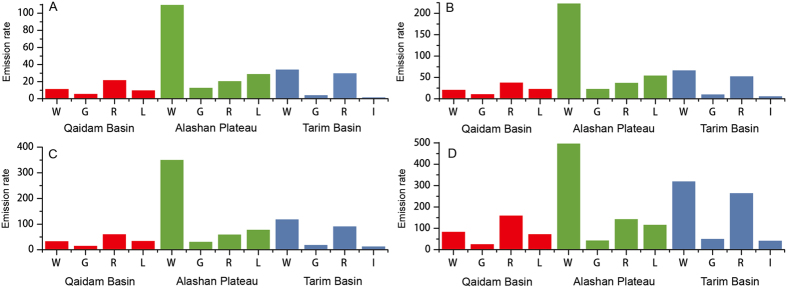
Availabilities of PM2.5 (**A**), PM5 (**B**), PM10 (**C**), and PM50 (**D**) (g m^−2^) during one aeolian event for different landscapes. W: wadi; G: gobi; R: riverbed; L: lakebed; and I: interdune.

The Qaidam Basin is a potential source of the Loess Plateau during Pleistocene^45^. Landscapes with higher modern dust aerosol availabilities in the Qaidam Basin include riverbeds, wadis and lakebeds (Fig. 2). In the Ala Shan Plateau and Tarim Basin, wadis have relatively higher modern dust aerosol availabilities than other landscapes. Due to extremely low contents of fine fractions (i.e., fractions <50 µm in diameter) in mobile sands, there are nearly no or extremely low fine fractions (i.e., <50 µm in diameter) in the transported materials obtained using collection sampler (*S2*), which indicates that the mobile sand dunes are not the dominant landscape for modern dust aerosol availability. However, in the other regions such as Sahara Desert and western Queensland, aeolian abrasion among sand grains breaks the clay coatings on grain surfaces of the dune sands, which act as the main source of particulate matter less than 10 microns (PM10)^46, 47^.

**Figure 3:**
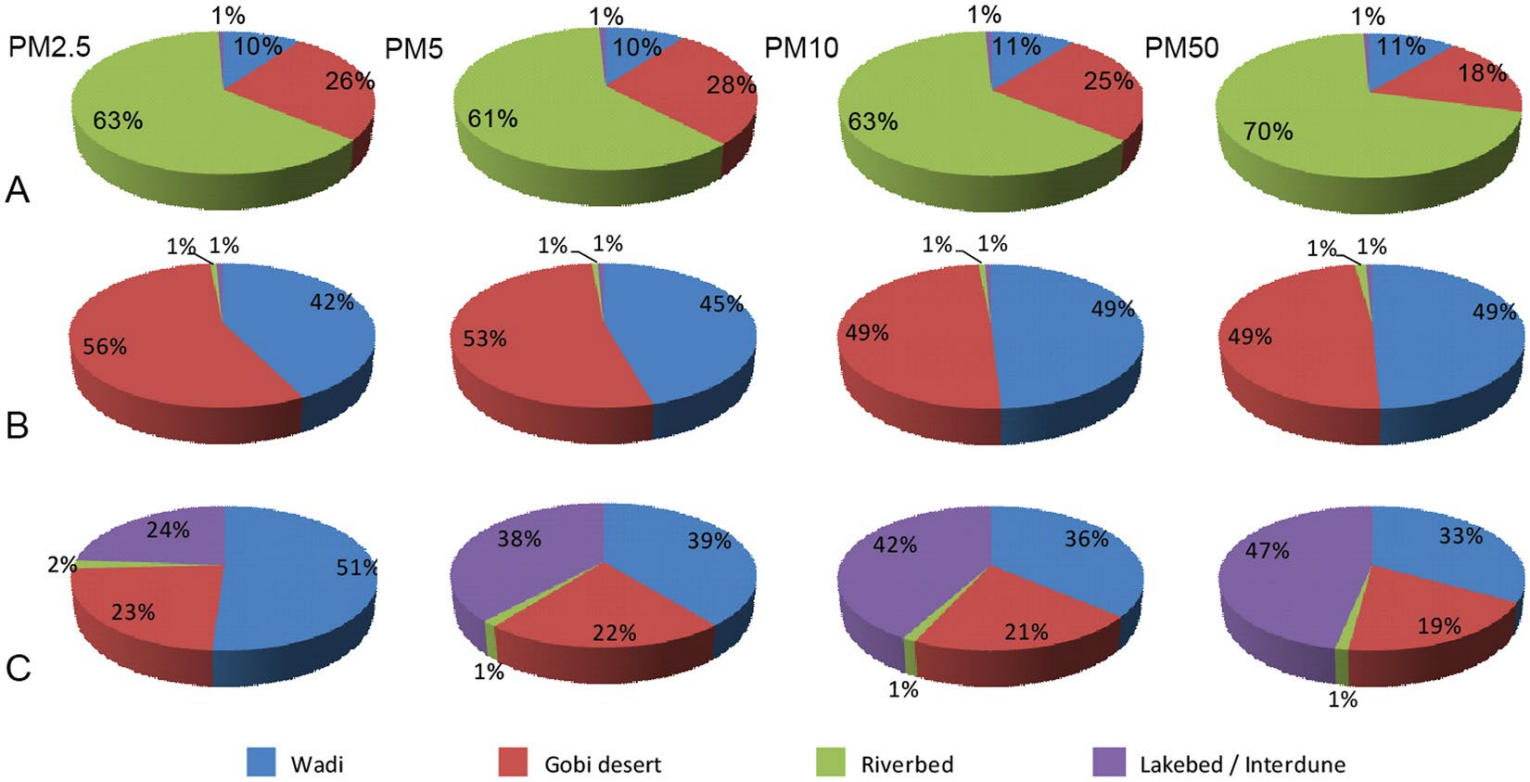
The comparison in the availabilities of PM2.5, PM5, PM10 and PM50 among different landscapes. (**A**) Qaidam Basin; (**B**) Ala Shan Plateau; and (**C**) Tarim Basin.

Except for the differences in the dust availability under the same landscape, there are variations in the dust aerosol availability among different landscapes (*S2*). After combining the areas of different landscapes, the results show that more than 60% of PM2.5 to PM50 fractions in the Qaidam Basin is emitted from riverbeds, while the gobi provides only 18–28% in the region (Fig. 3). In sharp contrast to the Qaidam Basin, only 1% of the dust originated from riverbeds in the Ala Shan and Tarim Basin, while the gobi and wadi provide 49–53% and 42–49% of the modern dust aerosol availabilities, respectively. In the Tarim Basin, more than 50% of the PM2.5 fractions are from wadis, while nearly half of the PM50 fractions are emitted from the interdunes located within the mobile sandy deserts.

Without considering the impacts of other regions of China, approximately 58% of the PM2.5 fractions may be generated on the Ala Shan Plateau and approximately 56% of the PM50 fractions may be generated on the Tarim Basin during a single high-intensity aeolian event in China (Table 1). Although the Qaidam Basin was proposed as the dominant dust source during the Quaternary Period for the Loess Plateau^45, 48^, the Qaidam Basin is unlikely to be a potential aeolian source for modern dust aerosols and loess sediment in East Asia because the percentages of PM2.5, PM5, PM10 and PM50 availabilities in the Qaidam Basin only occupied 14.04%, 12.16%, 11.49% and 12.77% in the three regions.

**Table 1:** Availabilities of PM2.5, PM5, PM10 and PM50 (Tg) generated in one heavy dust event in the Qaidam Basin, Ala Shan Plateau and Tarim Basin.

Region	PM2.5	PM5	PM10	PM50
Qaidam Basin	0.663	1.188	1.827	4.383
Alashan Plateau	2.723	5.177	7.545	10.647
Tarim Basin	1.337	3.407	6.533	19.301

Variation in modern dust aerosol availabilities for different landscapes may result in different modern dust aerosol emission responses to climate/environmental changes. In the Qadaim Basin, increases in precipitation, melted snow and glacial meltwater may promote sediment transport to riverbeds and consequently improve modern dust aerosol availabilities in the region. This suggests that global warming on the Qinghai-Tibet Plateau^49, 50^ may enhance modern dust aerosol availabilities. In contrast, for the Tarim Basin and Ala Shan Plateau, although environmental deterioration has resulted in most rivers and lakebeds drying up over the past few decades, there has been no significant change in the areas covered by wadis^51^, mobile sandy deserts and gobi compared with those during the Quaternary Period^52^. Hence, global warming may not trigger a large increase in erodible fine particles at the surface in these areas and consequently may not increase the modern dust aerosol availabilities in the above two regions. However, global warming may enhance the processes of physical, chemical and salt weathering^38, 53–56^ that occur on the surfaces of the gobi, wadis, riverbeds and lakebeds, which would increase modern dust aerosol availabilities.

Finally, more than 80% of modern dust aerosol availabilities in China are from the Ala Shan Plateau, Tarim Basin, and Qaidam Basin^23, 57–59^. According to meteorological records, there have been 2 to 5 annual dust storm events for the three regions from the early 21^st^ century to the present (*S3*). The annual modern dust aerosol availabilities calculated from dust storm events over the three regions for PM2.5, PM5, PM10 and PM50 are approximately 18, 36, 56, and 105 Tg, respectively *(S3*).Therefore, assuming that the magnitude of modern dust aerosol emissions is not overestimated by current dust emission models, the modern dust aerosol emissions in China are related to low-intensity aeolian processes that are below the minimum criteria of the WMO for classification as dust storm events.

## Conclusions

Although wadis, gobi, lakebeds, riverbeds, and interdunes are potential landscapes for modern dust aerosol emissions over the Tarim Basin, Qaidam Basin, and the Ala Shan Plateau, their roles in modern dust aerosol availabilities are different. In the Qaidam Basin, riverbeds play a major role in modern dust aerosol availabilities, while for the Ala Shan Plateau and Tarim Basin, wadis, gobi, and interdunes are the dominant landscapes for modern dust aerosol availability. In addition, the Qaidam Basin is not a major potential source of dust for sediment in the Loess Plateau and for modern dust aerosols transported to East Asia and North Pacific regions, at least in the context of modern aeolian processes.

## Methodology

### Study area

The sampling areas include the Tarim Basin, Ala Shan Plateau and Qaidam Basin with an area of 5,300,000, 2,700,000, and 121,000 km^2^, respectively, and are potential sources of modern dust aerosol emission in northwestern China (Fig. 1, Table 2). In these regions, the gobi, which is described as a “wide, shallow basin of which the smooth rocky bottom is filled with sand, silt or clay, pebbles or, more often, with gravel”^60, 61^, is extensively developed. In addition to the various landscapes, such as wadi (dry ancient lakebed /riverbed that contains water only during the periods of heavy rain or simply an intermittent stream), the gobi and interdune^37, 39^ that have high modern dust aerosol emissions, most water systems in these regions are temporarily developed, causing the riverbeds and lakebeds to dry up, providing abundant fine particles for emission via aeolian processes^38^.

**Table 2:** Areas of the Qaidam Basin, Ala Shan Plateau, and Tarim Basin and areas of landscapes with potential modern dust aerosol emission in the region.

Area (km^2^)	Qaidam Basin	Ala Shan Plateau	Tarim Basin
Total	121,000^73^	270,000^74^	530,000^51^
Wadi	5850^75^	10581.3^76^	20,000^77^
Gobi	31,000^78^	120,000^78^	73,000^78^
Riverbed	19,200^79^	1025.14^80^	856.98^81–83^
Lakebed	460^75^	755.92^84^	/
Interdune	/	/	216,000^85^

Note: “/”: At present there are only a few lakebeds and interdunes developed in the Tarim Basin, Qaidam Basin and Ala Shan Plateau. Therefore, their areas are negligible.

### Field sampling

Only loose and fine particles on the surface (i.e., <2000 µm in diameter) can be transported by aeolian processes^62^ to provide sources for modern dust aerosol emission. In China, these loose and fine particles are typically generated via water erosion, aeolian abrasion and weathering^38, 54–56^ and loosely accumulate on land surfaces such as wadis, gobi, lakebeds, interdunes and riverbeds^39^. Because only surface materials with approximately 1–10 cm depth are affected by aeolian processes^63, 64^, we collected samples within 10 cm of the surface during field sampling in 2013 and 2014. For each landscape, five representative samples were collected, except for the gobi on the Ala Shan Plateau where 15 samples were collected. In total, ninety-five samples were employed for further wind tunnel experiments. The sampling criteria were as follows: geomorphologic characteristics of the sampling sites are typical landscapes within an area of 10 × 10 km^2^, the underlying surfaces have no vegetation cover, no biological or physical soil crusts are present, and no human disturbance is detected. At each site, the samples were collected in horizontal intervals of approximately 100 to 200 m. The particle size characteristic of each landscape is shown in Table 3.

**Table 3:** Content as a percentage (%) of total particle size for the surface samples collected.

Region	Particle size (µm)	<2.5	<5	<10	<50	<100	<200	<2000
Qaidam Basin	Wadi	4.95	9.04	15.36	52.43	80.75	96.56	100
	Gobi	2.58	4.42	6.24	12.19	26.10	56.90	100
	Riverbed	6.43	10.92	16.47	54.15	81.14	95.26	100
	Lakebed	1.53	2.95	4.39	9.56	17.72	59.49	100
	*Mobile sand	0.00	0.00	0.00	0.00	5.68	58.12	100
Tarim Basin	Wadi	3.93	7.47	17.54	70.22	91.04	99.57	100
	Gobi	2.35	4.47	7.62	19.85	33.18	50.19	100
	Riverbed	5.77	9.77	16.36	47.64	84.87	100.00	100
	Interdune	5.63	9.55	14.91	39.05	71.95	95.05	100
	*Mobile sand	0.00	0.00	0.00	0.00	9.01	57.42	100
Ala Shan Plateau	Wadi	11.06	21.56	40.30	92.41	95.99	97.31	100
	Gobi	4.10	6.81	9.28	14.02	26.47	55.18	100
	Riverbed	8.82	15.32	23.89	62.84	86.91	95.58	100
	Lakebed	3.87	7.24	10.54	17.36	29.95	60.57	100
	*Mobile sand	0.00	0.00	0.00	0.49	3.87	14.54	100

*Mobile sand samples for the wind tunnel experiments were collected from the crest or stoss slope of shifting sand dunes.

### Wind tunnel experiments

**Figure 4:**
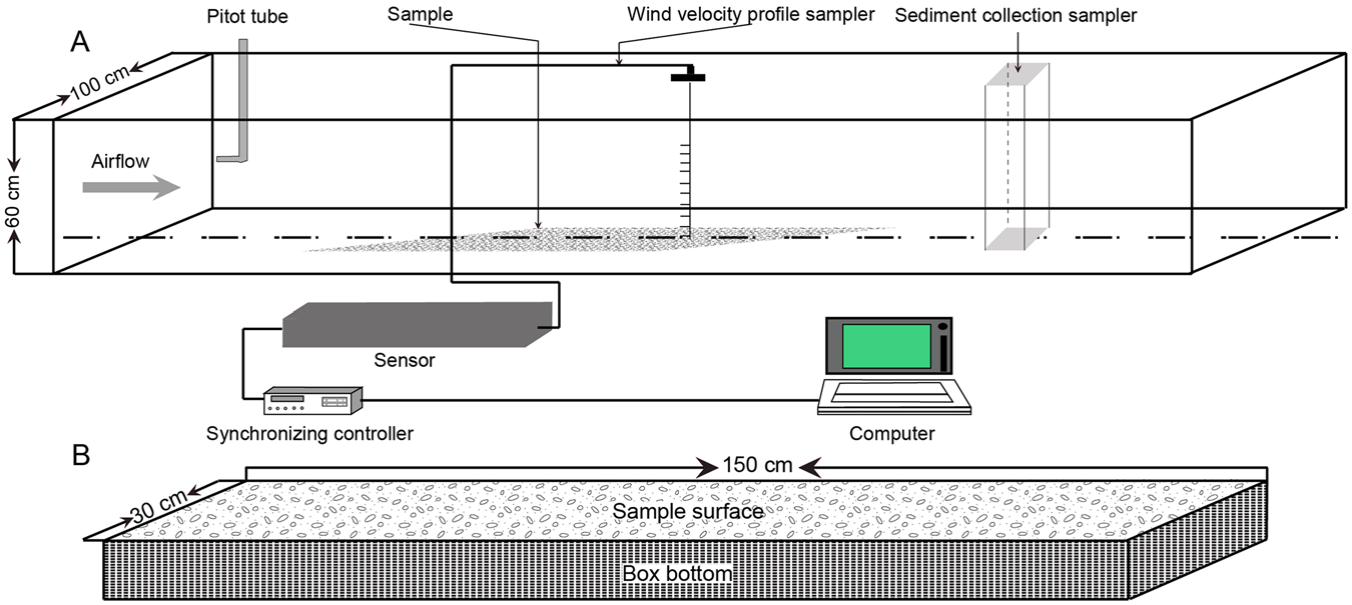
Schematic diagram of (**A**) the wind tunnel and sample arrangement during the wind tunnel experiment, and (**B**) the surface samples.

Wind tunnel experiments were performed at the Key Laboratory of Desert and Desertification in the Cold and Arid Regions Environmental and Engineering Research Institute, Chinese Academy of Sciences, China. The size and operating characteristics of the wind tunnel were described in detail in previous papers^65–68^, and the wind tunnel set-up is shown in Fig. 4.

Before the wind tunnel experiments, all samples were fully crushed and disaggregated. Each sample was spread over an area of 150 × 30 cm in the working section of the wind tunnel, with the surface at the same level as the bottom of the wind tunnel. At a distance of 30 cm downwind from the sample, a trap with 30 cm width and 30 cm height was used to collect windblown material.

According to the observational data from the weather stations in the study area from 1980 to 2014, the maximum wind velocity ranged from 14 to 22 m·s^−1^. Wind events with velocities of 5–10 m·s^−1^ occupied about 89–100% of those with velocities higher than the threshold wind velocity (5 m·s^−1^). During our preliminary experiments in November 2009, we found that, no erodible particles existed on the sample surfaces after approximately 360 seconds under a wind velocity of 8 m·s^−1^, and under a wind velocity of 22 m s^−1^, no erodible particles existed after 60 seconds. Hence, the free-stream wind velocity at the axis of the wind tunnel were set to 8, 10, 12, 14, 16, 18, 20, and 22 m·s^−1^. For each free-stream wind velocity, we simultaneously measured the wind velocity at 10 different heights (0.3, 0.6, 1.2, 2.4, 4.0, 8.0, 12.0, 16.0, 20.0, and 25.0 cm) above the floor of the wind tunnel using a wind profile sampler (Fig. 4).

We started the experiments with a free-stream wind velocity of 8 m s^−1^ until all erodible particles in the surface samples were completely eroded. Then, we repeated the experiments until the wind velocity reached 22 m·s^−1^. After all erodible particles in the surface samples were eroded at each wind velocity, the transported sediments collected by the sand trap were weighed and used for particle size analysis. Due to differences in atmospheric circulation^69, 70^, vegetation coverage^71^, precipitation, topography^39^, and subsequent transportation and sediment processes^72^, there may be variations in the magnitude of modern dust aerosol availability. Therefore, we discuss only the modern dust aerosol availability in a flat loose bed.

### Particle size analysis

After wind tunnel experiments have been finished, the transported and surface materials collected in the field were analyzed for particle size using a Mastersizer 2000 (Malvern Co. Ltd., Malvern, UK). Before conducting the particle size measurements, we immersed the sediments in 10% H_2_O_2_ followed by immersion in 12.7% HCl to remove any plant debris and disperse aggregates within the sediments. The sample residue was finally treated with 10 mL of 0.05 M (NaPO_3_)_6_ on an ultrasonic vibrator for 10 min to facilitate dispersion before measuring the particle size.

### Data processing

During the wind tunnel experiments with increasing wind velocities, the erodible particles were gradually depleted. Therefore, the raw data acquired during the wind tunnel experiments and during the measuring procedures must be re-processed using statistical methods. Stronger winds transport not only coarse materials but also materials that would be transported at lower velocities. Therefore, we used the weighted average method to determine the transports at the certain wind speed:

1 $${R}_{w}=({R}_{1}{M}_{1}+{R}_{2}{M}_{2}+\cdots +{R}_{i}{M}_{i}+\cdots +{R}_{m}{M}_{m})/({M}_{1}+{M}_{2}+\cdots +{M}_{i}+\cdots +{M}_{m})$$

where *R*_w_ is the weighted mean of different particle sizes, *R*_i_ is the raw data for the contents (%) of different particle sizes at predefined wind velocities (8 to 22 m·s^−1^), and *M*_i_ is the corresponding contents of the raw transported material at the same velocities. Combining the results of particle size analysis, the availabilities of PM2.5, PM5.0, PM10 and PM50 were acquired.

In natural conditions, the bonding abilities of particles, the cover of coarse fractions on the fine particles, and the differences in airflow structures^1^, may result in lower contents of loose and fine particles on intact surfaces than on disturbed samples. Therefore, the modern dust aerosol availabilities from the wind tunnel experiments may be higher than those in the field. In addition, meteorological records indicate that the wind velocity rarely exceeds 22 m s^−1^ during severe dust events. Therefore, we took the transport acquired at a velocity of 22 m·s^−1^ as the maximum modern dust aerosol availability in the study regions. Additional explanations and discussions are provided in *S2*.

## Electronic supplementary material

Supplementary Information
