# Supplementary material for: Modern dust aerosol availability in northwestern China
Source: Sci Rep. 2017 Aug 18;7:8741. doi: 10.1038/s41598-017-09458-w (PMC5562760; doi:10.1038/s41598-017-09458-w)
Supplement: Supplementary file 1 — Supplementary Information [file 41598_2017_9458_MOESM1_ESM.doc]

# Modern dust aerosol availability in northwestern China

**^1, 2^Xunming Wang^*^, ^3^Hong Cheng, ^4^****Huizheng Che, ^5^****Jimin Sun, ^6^****Huayu Lu, ^7^Mingrui Qiang, ^8^Ting Hua, ^1^Bingqi Zhu, ^8^Hui Li, ^1^Wenyong Ma, ^1^Lili Lang, ^1^Linlin Jiao, ^1^Danfeng Li**

^1^Key Laboratory of Water Cycle and Related Land Surface Processes, Institute of Geographic Sciences and Natural Resources Research, Chinese Academy of Sciences, Beijing 100101, China

^2^University of Chinese Academy of Sciences, Beijing 100049, China

^3^State Key Laboratory of Earth Surface Processes and Resource Ecology, Beijing Normal University, Beijing 100875, China

^4^State Key Laboratory of Severe Weather (LASW), Institute of Atmospheric Composition, Chinese Academy of Meteorological Sciences, Beijing 100081, China

^5^Key Laboratory of Cenozoic Geology and Environment, Institute of Geology and Geophysics, Chinese Academy of Sciences, Beijing 100029, China

^6^School of Oceanographic and Geographic Sciences, Nanjing University, Nanjing 210023, China

^7^Key Laboratory of Western China’s Environmental Systems (Ministry of Education), College of Earth and Environmental Sciences, Lanzhou University, Lanzhou 73000, China

^8^Key Laboratory of Desert and Desertification, Cold and Arid Regions Environmental and Engineering Research Institute, Chinese Academy of Sciences, Lanzhou 730000, China

* Corresponding author: E-mail: xunming@igsnrr.ac.cn

**Supplementary Information**

**S1 Uncertainties of modern dust aerosol emission modeling and of its availabilities**

Modern dust aerosol emissions in different fractions have been estimated using numeric models^20, 23, 25, 86, 87, 88, 89^ by considering complex nonlinear processes that are governed by meteorology and the state properties of land surfaces^15, 90^ at both global and regional scales. Due to the physical parameterizations derived for different conditions and the lack of critical data (i.e., dry-sieved size distribution and aeolian surface roughness), only simple schemes that often employ empirical parameterizations of the vertical flux of emitted mineral aerosols in terms of the cubed or quadratic surface wind speed and the uniformly fixed threshold friction velocity^91^ have been employed in the modern dust aerosol emission models. However, the physically parameterized and the threshold friction velocity vary in space and time due to soil moisture variation, surface roughness heterogeneity and cyclic and seasonal changes in vegetation^86, 89, 90^. Furthermore, there are large differences of modern dust aerosol emissions for different models^16, 90^, and uncertainty estimates should be emphasized to focus on critical processes^86^. In addition, the temporal and spatial sampling scales of the models are usually coarser than the observations^21^.

After fine particles are generated by aeolian processes, they can be regarded as modern dust aerosol availabilities of the source regions. However, not all modern dust aerosol availabilities are emitted outside from their source areas. For example, approximately 30%^22^, 70-90%^92^ and 37-63%^93^ of the fractions emitted from the source regions are deposited in situ. Although there are uncertainties in determining modern dust aerosol emission rates, the variation of modern dust aerosol availabilities indicated the trends of modern dust aerosol emissions in different geomorphologic settings and regions^79, 80, 81^. Therefore, it is crucial to evaluate the variation in modern dust aerosol availabilities from different landscapes in China.

**S2 Availabilities of PM2.5, PM5, PM10 and PM50 in different landscapes**

There are extremely low contents of fine fractions (i.e., <50 µm in diameter) for surface mobile sands (Table 3) and transported sands (Table S1). In the subsequent estimation for the modern dust aerosol availabilities, we do not discuss those generated from mobile sand surfaces. Within the Tarim Basin, however, interdunes with high contents of fine fractions are common among the mobile dunes^94^ and are also the main landscape for modern dust aerosol emissions^26^. Therefore, the modern dust aerosol availabilities in interdunes are also included in this study. Although some studies^95, 96^ have considered that the degraded coppice dunes developed in arid China may provide abundant fine fractions for dust emissions, there are very low intensities of aeolian processes on their surfaces due to the high vegetation cover (>14%)^97, 98^. Therefore, we concluded that the degraded coppice dunes may contribute low-level dust emissions over a broad area and more researches are needed in the near future.

**Table S1.** The experimental percent (%) of PM2.5, PM5.0, PM10 and PM50 in transported materials.

| Region | Landscape | PM2.5 | PM5 | PM10 | PM50 |
| --- | --- | --- | --- | --- | --- |
| Qaidam Basin | Wadi | 3.19 | 5.67 | 8.96 | 26.83 |
|  | Mobile sand | 0.00 | 0.00 | 0.00 | 0.00 |
|  | Gobi | 1.26 | 2.42 | 3.41 | 6.88 |
|  | Riverbed | 7.34 | 13.06 | 21.21 | 61.26 |
|  | Lakebed | 0.90 | 1.95 | 2.89 | 7.83 |
| Ala Shan Plateau | Wadi | 12.43 | 25.59 | 41.54 | 82.02 |
|  | Mobile sand | 0.00 | 0.00 | 0.00 | 0.06 |
|  | Gobi | 2.55 | 4.52 | 6.14 | 8.48 |
|  | Riverbed | 8.90 | 16.49 | 26.50 | 73.53 |
|  | Lakebed | 4.77 | 8.95 | 12.79 | 21.75 |
| Tarim Basin | Wadi | 4.88 | 9.50 | 16.94 | 56.10 |
|  | Mobile sand | 0.00 | 0.00 | 0.00 | 0.00 |
|  | Gobi | 0.62 | 1.46 | 2.69 | 9.04 |
|  | Riverbed | 4.47 | 7.91 | 13.73 | 47.19 |
|  | Interdune | 0.10 | 0.39 | 0.82 | 3.61 |

The wind tunnel experiment results show that there are high differences for dust aerosol availability even within the same landscape. For example, the coefficients of variation (the ratios of the standard deviations to the means) for the dust aerosol availabilities of the wadi, gobi desert, riverbed and the lakebed are 0.92, 0.56, 0.14, 0.34, and 0.95, respectively (Table S2). Therefore, in the present study, we only took the average value as the intensity of dust aerosol availability of each landscape.

**Table S2.** The coefficients of variation for the dust aerosol availability of different landscapes in the Qaidam Basin, Ala Shan Plateau, and the Tarim Basin.

| Region | Landscapes | PM2.5 | PM5 | PM10 | PM50 |
| --- | --- | --- | --- | --- | --- |
| Qaidam Basin | Wadi | 0.92 | 0.78 | 0.71 | 0.42 |
|  | Gobi | 0.56 | 0.52 | 0.50 | 0.45 |
|  | Riverbed | 0.14 | 0.14 | 0.16 | 0.15 |
|  | Lakebed | 0.34 | 0.19 | 0.18 | 0.19 |
| Ala Shan Plateau | Wadi | 0.95 | 0.94 | 0.92 | 0.84 |
|  | Gobi | 0.39 | 0.35 | 0.35 | 0.33 |
|  | Riverbed | 0.26 | 0.22 | 0.21 | 0.30 |
|  | Lakebed | 0.50 | 0.48 | 0.47 | 0.41 |
| Tarim Basin | Wadi | 0.48 | 0.51 | 0.50 | 0.45 |
|  | Gobi | 0.96 | 0.68 | 0.62 | 0.53 |
|  | Riverbed | 0.27 | 0.26 | 0.24 | 0.17 |
|  | Interdune | 0.53 | 0.35 | 0.32 | 0.20 |

Theoretically, any aeolian processes may result in the transport of fine particles in the region. Considering that the occurrence of severe dust storm events play important roles in modern dust aerosol emissions in China^99^, we took the fine fraction contents acquired under a wind velocity of 22 m·s^-1^ in the wind tunnel experiments as the modern dust aerosol availabilities under severe dust storm event. The modern dust aerosol availabilities for different landscapes under a wind velocity of 22 m·s^-1^ are shown in Table S3. Under severe dust storm conditions in the field, the modern dust aerosol availabilities vary on the different landscape surface.

**Table S3. Modern dust aerosol availabilities (Tg) for different landscapes.**

| Region | Landscape | PM2.5 | PM5 | PM10 | PM50 |
| --- | --- | --- | --- | --- | --- |
| Qadaim Basin | Wadi | 0.066 | 0.122 | 0.194 | 0.490 |
|  | Gobi | 0.173 | 0.330 | 0.464 | 0.794 |
|  | Riverbed | 0.419 | 0.725 | 1.154 | 3.066 |
|  | Lakebed | 0.004 | 0.011 | 0.016 | 0.033 |
| Ala Shan Plateau | Wadi | 1.161 | 2.362 | 3.704 | 5.250 |
|  | Gobi | 1.519 | 2.736 | 3.721 | 5.164 |
|  | Riverbed | 0.021 | 0.038 | 0.061 | 0.146 |
|  | Lakebed | 0.022 | 0.041 | 0.059 | 0.088 |
| Tarim Basin | Wadi | 0.683 | 1.332 | 2.372 | 6.393 |
|  | Gobi | 0.309 | 0.733 | 1.343 | 3.654 |
|  | Riverbed | 0.025 | 0.045 | 0.078 | 0.226 |
|  | Interdune | 0.320 | 1.298 | 2.740 | 9.029 |

**S3 Significance of heavy dust events for modern dust aerosol emission in the Qaidam Basin, Ala Shan Plateau, and Tarim Basin**

According to the classification criteria of the China Meteorological Administration (CMA), severe and weak dust storm events are recorded under the conditions of an extreme wind velocity of ≥17 m·s^-1^ and visibility of ≤500 m and of an extreme wind velocity of ≥10 m·s^-1^ and visibility of ≤1000 m, respectively. The modern dust aerosol availabilities during each single severe dust event in the Qadaim Basin, Ala Shan Plateau, and Tarim Basin are shown in Table 1. The spatial distribution of the CMA meteorological stations for the Ala Shan Plateau, Tarim Basin and Qaidam Basin is shown in Figure S1. There are annual fluctuations in severe dust event occurrences over the Ala Shan Plateau, the Tarim Basin and Qaidam Basin from 1980 to 2014 in Figure S2, which suggests that there have been plenty of modern dust aerosol emissions over the Ala Shan Plateau than over the Qaidam Basin and Tarim Basin in the past three decades. In addition, from 2000 to 2014, the annual frequencies of severe dust events in the three regions are 5, 3, and 2 times, respectively. Combining the results of modern dust aerosol availabilities of different landscapes acquired in wind tunnel experiments, the annual modern dust aerosol availabilities from severe modern dust aerosol events in these three regions may not exceed 18, 36, 56, and 105 Tg for PM2.5, PM5, PM10 and PM50, respectively.


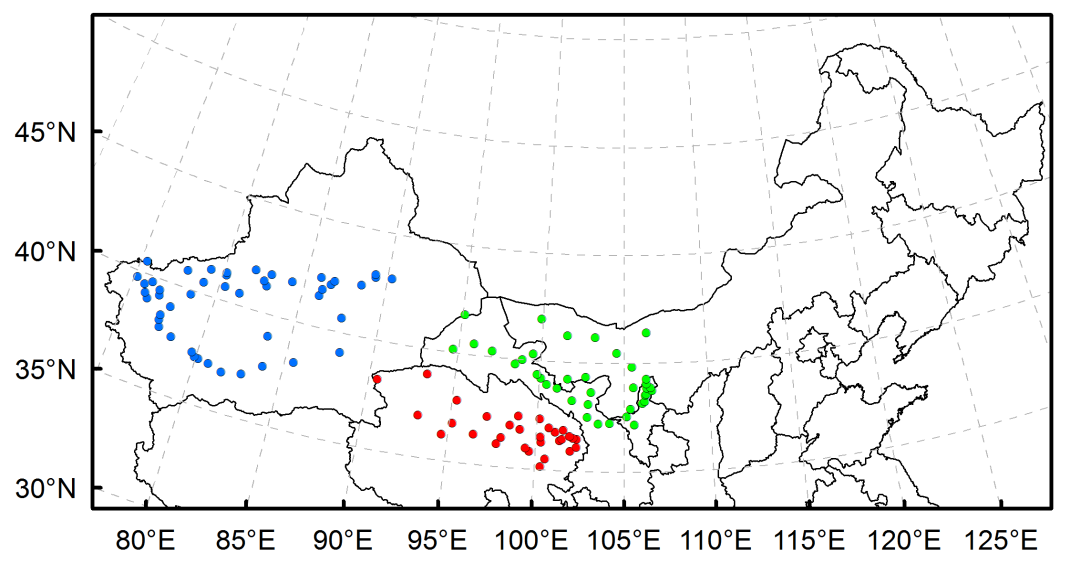


**Figure S1.** Spatial distributions of the CMA meteorological stations for the Ala Shan Plateau (Green dot), Tarim Basin (Blue dot) and Qaidam Basin (Red dot). The figure was finished using Arcgis software (version 10.1, ESRI Inc., Redlands, California, USA), which can be downloaded from the internal network of Institute of Geographic Sciences and Natural Resources Research, Chinese Academy of Sciences.


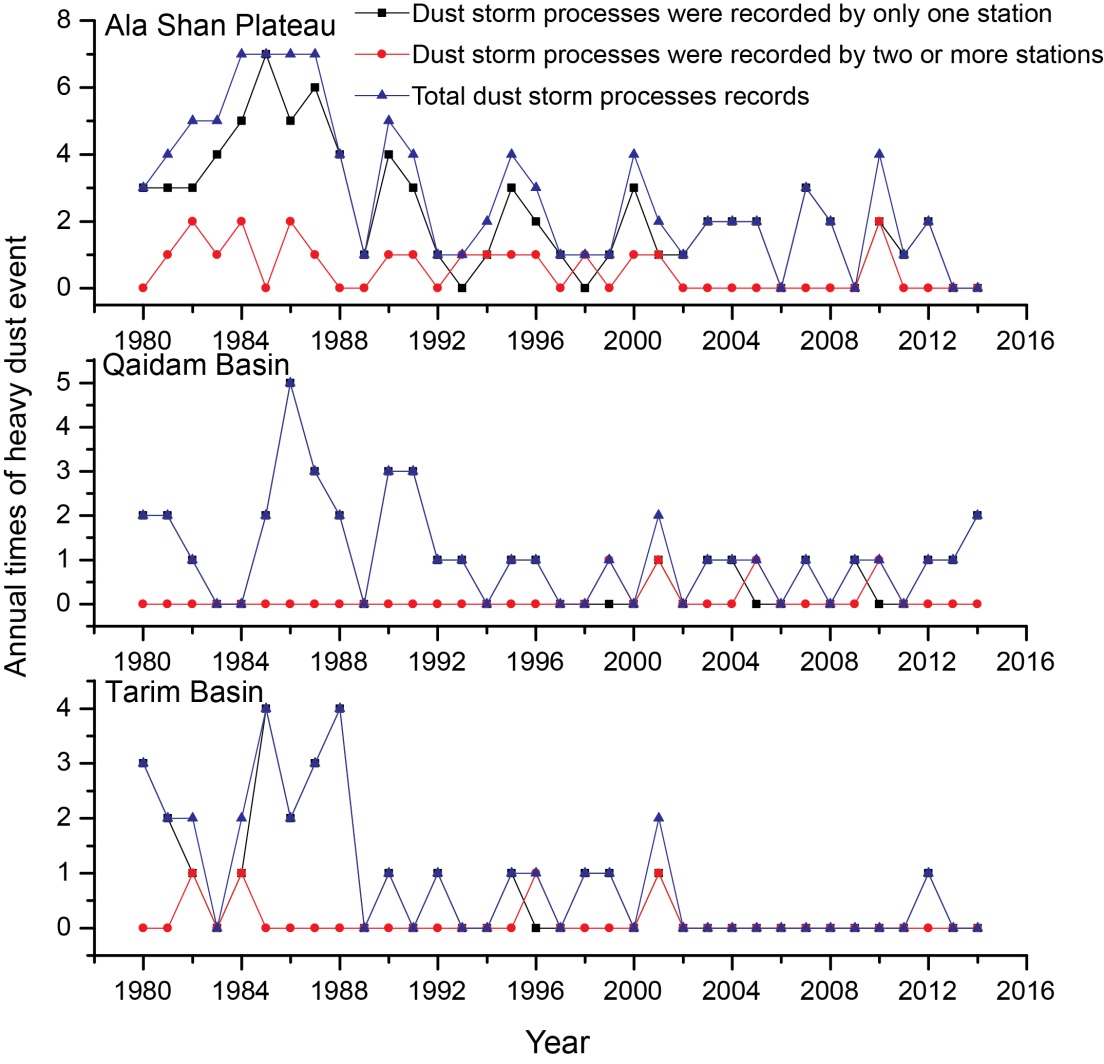


**Figure S2.** Annual trends in severe dust storm processes (extreme wind velocity of ≥17 m·s^-1^ and the visibility of ≤500 m) over the Ala Shan Plateau, Qaidam Basin, and Tarim Basin.

The modern dust aerosol availabilities in these regions may be overestimated. The main reasons include the following several parts. First, for a single severe dust event, although it may be recorded by one or more weather stations, it is impossible to cover the entire area (i.e., the entire Ala Shan Plateau). Second, over the past three decades, besides the occurrence of severe dust events, there have been weaker dust events with low-intensity modern dust aerosol emissions occurring in the region. For example, from 2000 to 2014, the maximum annual frequency of weak dust events over the Ala Shan Plateau, Qaidam Basin, and Tarim Basin were 18, 5, and 14 times (Figure S3). It is difficult to determine the total modern dust aerosol availabilities under weak dust event conditions. However, if the contents of PM2.5, PM5, PM10, and PM50 under a wind velocity of 14 m·s^-1^ are regarded as proxies for the modern dust aerosol availabilities during weak dust events, the values for the total modern dust aerosol availabilities range from 5.24% to 24.11% under a wind velocity of 22 m·s^-1^ (Table S4). Therefore, although most aeolian processes may not result in dust events that meet the criteria for dust storm records, they also play key roles in modern dust aerosol emissions and consequently on regional and global climate and environmental changes.


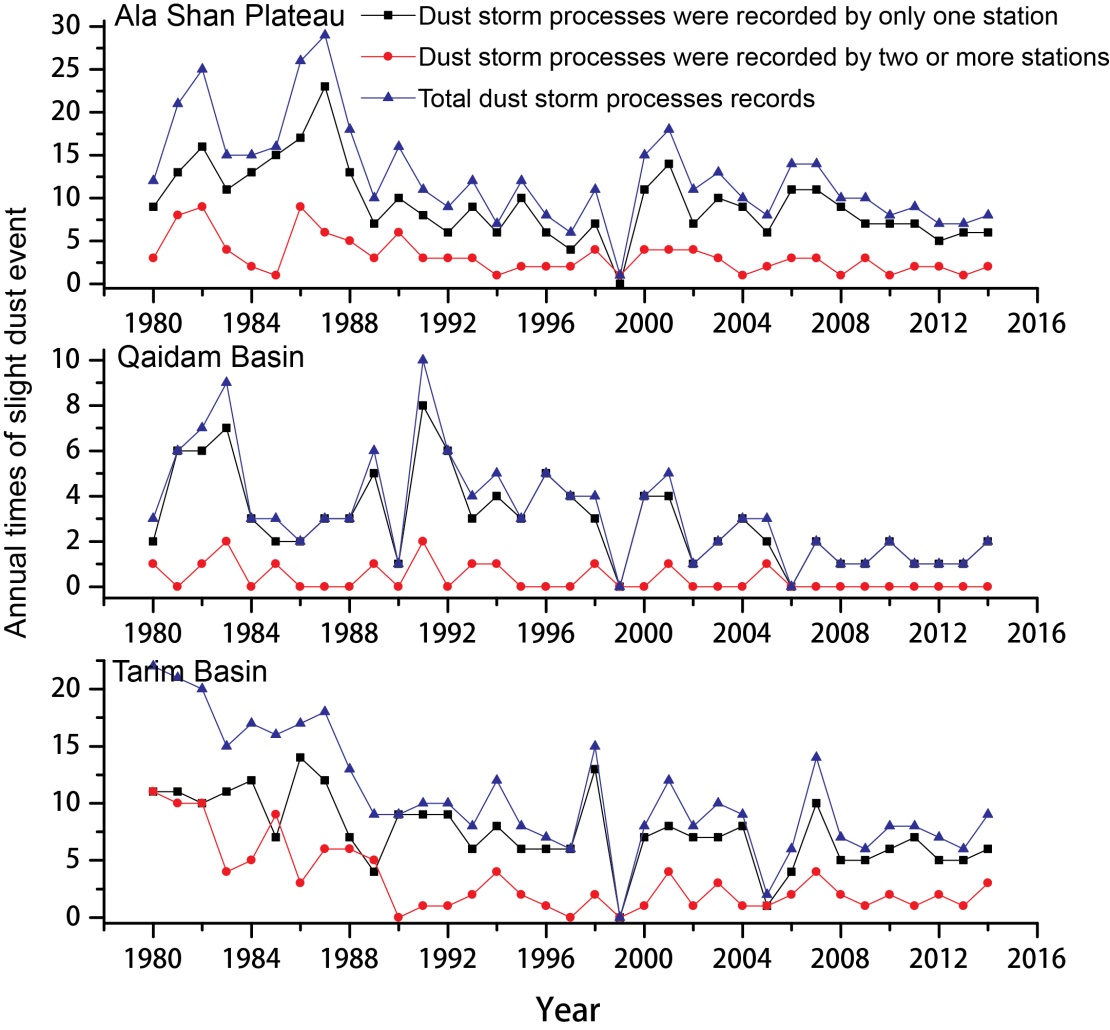


**Figure S3.** Annual trends in weak dust storm processes (with an extreme wind velocity of ≥ 10 m·s^-1^ and visibility of ≤ 1000 m) over the Ala Shan Plateau, Qaidam Basin, and Tarim Basin.

**Table S4.** Percentage (%) of the PM2.5, PM5, PM10, and PM50 contents under weak dust events (with a wind velocity below 14 m·s^-1^) in comparison to those with a wind velocity below 22 m·s^-1^.

| Region | Landscape | PM2.5 | PM5 | PM10 | PM50 |
| --- | --- | --- | --- | --- | --- |
| Qaidam Basin | Riverbed | 16.58 | 15.92 | 15.95 | 16.46 |
|  | Gobi desert | 13.32 | 13.12 | 13.17 | 13.04 |
|  | Lakebed | 12.79 | 14.99 | 15.56 | 15.71 |
| Ala Shan Plateau | Wadi | 5.68 | 5.38 | 5.27 | 5.24 |
|  | Riverbed | 9.78 | 9.45 | 9.42 | 9.43 |
|  | Lakebed | 6.36 | 6.29 | 6.38 | 6.31 |
| Tarim Basin | Riverbed | 7.80 | 7.77 | 7.66 | 7.56 |
|  | Interdune | 24.11 | 21.04 | 19.95 | 16.55 |
|  | Gobi desert | 12.34 | 10.36 | 10.05 | 9.58 |
|  | Wadi | 7.31 | 7.37 | 7.42 | 7.21 |

**References**

1. Mahowald, N. M. *et al*. Atmospheric global dust cycle and iron inputs to the ocean. *Global Biogeochem. Cycles.* **19**, GB4025 (2005).
2. Cakmur, R. V. *et al*. Constraining the magnitude of the global dust cycle by minimizing the difference between a model and observations. *J. Geophys. Res.* **111** (D06), 207 (2006).
3. Uno, I. *et al*. Dust model intercomparison (DMIP) study over Asia: overview. *J. Geophys. Res*. **111**(D12), 213 (2006).
4. Xi, X. & Sokolik, I. N. Seasonal dynamics of threshold friction velocity and dust emission in Central Asia. *J. Geophys. Res. Atmos.* **120**, 1536–1564 (2015).
5. Darmenova, K., Sokolik, I. N., Shao, Y., Marticorena, B. & Bergametti, G. Development of a physically based dust emission module within the Weather Research and Forecasting (WRF) model: Assessment of dust emission parameterization and input parameters for source regions in Central and East Asia. *J. Geophys. Res.* **114** (D14), 201 (2009).
6. Darmenova, K. & Sokolik, I. N. Assessing uncertainties in dust emission in the Aral Sea region caused by meteorological fields predicted with a mesoscale model. *Global Planet. Change.* **56**, 297–310 (2007).
7. Chen, S. *et al*. Regional modeling of dust mass balance and radiative forcing over East Asia using WRF-Chem. *Aeolian Res*. **15**, 15–30 (2014).
8. Zhao, T., Gong, S., Zhang, X. & McKendry, I. G. Modeled size-segregated wet and dry deposition budgets of soil dust aerosol during ACE-Asia 2001: Implications for trans-Pacific transport. *J. Geophys. Res. Atmos.* **108**(D23), 8665 (2003).
9. Wang, X., Dong, Z., Zhang, J., Qu, J. & Zhao, A. Grain size characteristics of dune sands in the central Taklimakan Sand Sea. *Sediment. Geol.* **161**, 1–14 (2003).
10. Wang, X., Dong, Z., Yan, P., Yang, Z. & Hu, Z. Surface sample collection and dust source analysis in northwestern China. *Catena* **59**, 35–53 (2005).
11. Sweeney, M. R. *et al*. Sand dunes as potential sources of dust in northern China. *Sci. China Earth Sci.* **59**, 760–769 (2016).
12. Wiggs, G. F. S., Thomas, D. S. G., Bullard, J. E. & Livingstone, I. Dune mobility and vegetation cover in the southwest Kalahari Desert. *Earth Surf. Proc. Land.* **20**, 515–530 (1995).
13. Wiggs, G. F. S., Livingstone, I., Thomas, D. S. G. & Bullard, J. E. Airflow and roughness characteristics over partially vegetated linear dunes in the southwest Kalahari Desert. *Earth Surf. Proc. Land.* **21**, 19–34 (1996).
14. Zhou, Z. & Zhang, G. Typical severe dust storms in northern China during 1954-2002. *Chin. Sci. Bull.* **48**, 2366–2370 (2003).

**Table S1.** The experimental percent (%) of PM2.5, PM5, PM10 and PM50 in transported materials.

| Region | Landscape | PM2.5 | PM5 | PM10 | PM50 |
| --- | --- | --- | --- | --- | --- |
| Qaidam Basin | Wadi | 3.19 | 5.67 | 8.96 | 26.83 |
|  | Mobile sand | 0.00 | 0.00 | 0.00 | 0.00 |
|  | Gobi | 1.26 | 2.42 | 3.41 | 6.88 |
|  | Riverbed | 7.34 | 13.06 | 21.21 | 61.26 |
|  | Lakebed | 0.90 | 1.95 | 2.89 | 7.83 |
| Ala Shan Plateau | Wadi | 12.43 | 25.59 | 41.54 | 82.02 |
|  | Mobile sand | 0.00 | 0.00 | 0.00 | 0.06 |
|  | Gobi | 2.55 | 4.52 | 6.14 | 8.48 |
|  | Riverbed | 8.90 | 16.49 | 26.50 | 73.53 |
|  | Lakebed | 4.77 | 8.95 | 12.79 | 21.75 |
| Tarim Basin | Wadi | 4.88 | 9.50 | 16.94 | 56.10 |
|  | Mobile sand | 0.00 | 0.00 | 0.00 | 0.00 |
|  | Gobi | 0.62 | 1.46 | 2.69 | 9.04 |
|  | Riverbed | 4.47 | 7.91 | 13.73 | 47.19 |
|  | Interdune | 0.10 | 0.39 | 0.82 | 3.61 |

**Table S2.** The coefficients of variation (the ratios of the standard deviations to the means) for the dust aerosol availability of each landscape in the Qaidam Basin, Ala Shan Plateau, and the Tarim Basin.

| Region | Landscape | PM2.5 | PM5 | PM10 | PM50 |
| --- | --- | --- | --- | --- | --- |
| Qaidam Basin | Wadi | 0.92 | 0.78 | 0.71 | 0.42 |
|  | Gobi | 0.56 | 0.52 | 0.50 | 0.45 |
|  | Riverbed | 0.14 | 0.14 | 0.16 | 0.15 |
|  | Lakebed | 0.34 | 0.19 | 0.18 | 0.19 |
| Ala Shan Plateau | Wadi | 0.95 | 0.94 | 0.92 | 0.84 |
|  | Gobi | 0.39 | 0.35 | 0.35 | 0.33 |
|  | Riverbed | 0.26 | 0.22 | 0.21 | 0.30 |
|  | Lakebed | 0.50 | 0.48 | 0.47 | 0.41 |
| Tarim Basin | Wadi | 0.48 | 0.51 | 0.50 | 0.45 |
|  | Gobi | 0.96 | 0.68 | 0.62 | 0.53 |
|  | Riverbed | 0.27 | 0.26 | 0.24 | 0.17 |
|  | Interdune | 0.53 | 0.35 | 0.32 | 0.20 |

**Table S3. Modern dust aerosol availabilities (Tg) for different landscapes.**

| Region | Landscape | PM2.5 | PM5 | PM10 | PM50 |
| --- | --- | --- | --- | --- | --- |
| Qadaim Basin | Wadi | 0.066 | 0.122 | 0.194 | 0.490 |
|  | Gobi | 0.173 | 0.330 | 0.464 | 0.794 |
|  | Riverbed | 0.419 | 0.725 | 1.154 | 3.066 |
|  | Lakebed | 0.004 | 0.011 | 0.016 | 0.033 |
| Ala Shan Plateau | Wadi | 1.161 | 2.362 | 3.704 | 5.250 |
|  | Gobi | 1.519 | 2.736 | 3.721 | 5.164 |
|  | Riverbed | 0.021 | 0.038 | 0.061 | 0.146 |
|  | Lakebed | 0.022 | 0.041 | 0.059 | 0.088 |
| Tarim Basin | Wadi | 0.683 | 1.332 | 2.372 | 6.393 |
|  | Gobi | 0.309 | 0.733 | 1.343 | 3.654 |
|  | Riverbed | 0.025 | 0.045 | 0.078 | 0.226 |
|  | Interdune | 0.320 | 1.298 | 2.740 | 9.029 |

**Table S4.** Percentage (%) of the PM2.5, PM5, PM10, and PM50 contents under weak dust events (with a wind velocity below 14 m·s^-1^) in comparison to those with a wind velocity below 22 m·s^-1^.

| Region | Landscape | PM2.5 | PM5 | PM10 | PM50 |
| --- | --- | --- | --- | --- | --- |
| Qaidam Basin | Riverbed | 16.58 | 15.92 | 15.95 | 16.46 |
|  | Gobi desert | 13.32 | 13.12 | 13.17 | 13.04 |
|  | Lakebed | 12.79 | 14.99 | 15.56 | 15.71 |
| Ala Shan Plateau | Wadi | 5.68 | 5.38 | 5.27 | 5.24 |
|  | Riverbed | 9.78 | 9.45 | 9.42 | 9.43 |
|  | Lakebed | 6.36 | 6.29 | 6.38 | 6.31 |
| Tarim Basin | Riverbed | 7.80 | 7.77 | 7.66 | 7.56 |
|  | Interdune | 24.11 | 21.04 | 19.95 | 16.55 |
|  | Gobi desert | 12.34 | 10.36 | 10.05 | 9.58 |
|  | Wadi | 7.31 | 7.37 | 7.42 | 7.21 |
